# Reexamining Multisystem Desmoid Tumors Linked to Gardner's Syndrome: A Clinical Case

**DOI:** 10.1155/crgm/6882566

**Published:** 2025-02-28

**Authors:** Haijia Zhang, Yongjie Wu, Xiushan Dong, Jie An

**Affiliations:** General Surgery Department, Shanxi Bethune Hospital, Shanxi Academy of Medical Sciences, Tongji Shanxi Hospital, Third Hospital of Shanxi Medical University, Taiyuan 030000, China

**Keywords:** desmoid tumor (DT), familial adenomatous polyposis (FAP), Gardner's syndrome (GS)

## Abstract

A variation of familial adenomatous polyposis (FAP), known as Gardner's syndrome (GS), can manifest as extraintestinal tumors, such as desmoid tumors (DTs). A key part in the diagnosing process is played by the clinician. Because DT frequently occurs before intestinal polyposis develops, doctors can identify the underlying illness early by looking for DT's telltale signs. Nevertheless, in this instance, the patient's failure to recognize the illness promptly following the excision of the tumor in the abdomen wall caused a delay in the diagnosis and impacted the disease's course.

## 1. Introduction

Familial adenomatous polyposis (FAP) (OMIM #175100) described as a result of mutations of the adenomatous polyposis coli (APC) gene (OMIM #152230) have an autosomal dominant pattern of inheritance. Gardner's syndrome (GS) (OMIM #175100) is a variant of FAP, and the term used to describe the clinical features consists of colonic polyps, osteomas, and both cutaneous and subcutaneous soft tissue tumors. Desmoid tumors (DTs) (OMIM #129630) located in the abdominal wall and intra-abdominally occur to at least 10%–15% of patients with FAP [1, 2]. It has been shown that point mutations in the APC gene on chromosome 5q21 can cause both FAP and GS [3]. Features of the abdominal wall are often detected before intestinal polyposis. Identification of the features of GS can therefore serve as a sensitive marker of this condition, facilitating early referral, diagnosis, and instigation of life-saving treatment.

## 2. Case Report

**Figure 1:**
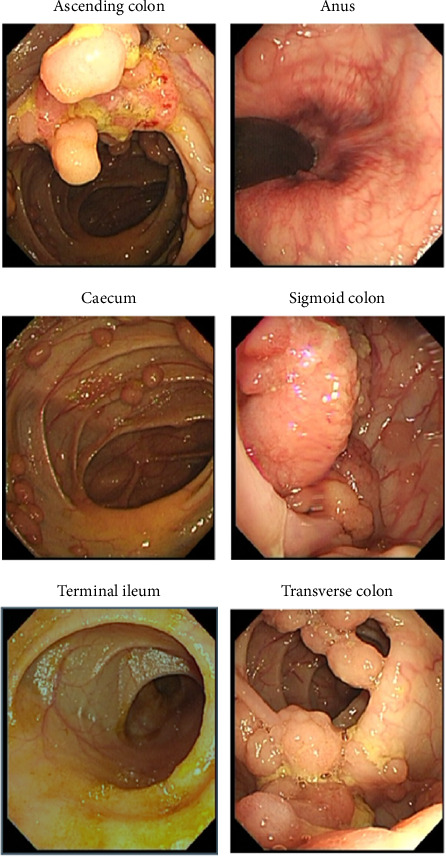
Colonoscopy report shows multiple colorectal polyps.

**Figure 2:**
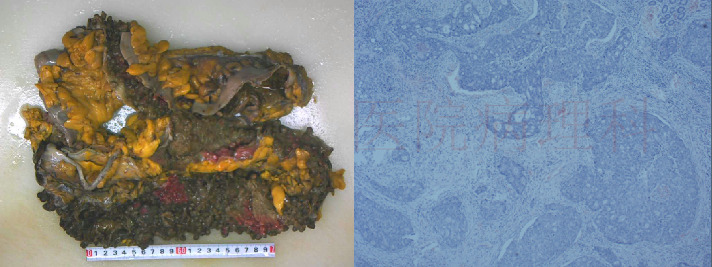
Postoperative pathology showed canceration (hematoxylin and eosin stain; original magnification, 40x).

The patient, a 32-year-old woman, was admitted for closure operation 9 months after ileostomy surgery. Nine months ago, the patient had diarrhea without obvious cause, 2-3 times a day, with yellow watery stool and occasional mucous and bloody stool and a feeling of incomplete defecation. Colonoscopy showed multiple sessile polyps of varying sizes ranging from 0.3–3.5 cm in the colon and rectum (Figure 1). Biopsy showed high-grade dysplasia and adenocarcinoma in the ascending colon and sigmoid colon glands (Figure 2). The patient underwent total colectomy and proctocolectomy with ileal pouch-anal anastomosis (IPAA) and ileostomy under general anesthesia. The postoperative pathological results, along with genetic testing that identified one point mutation in the APC gene (c.3183_3187delACAA/p.Gln1062∗), collectively indicated a diagnosis of FAP with carcinoma. Subsequently, the patient received four cycles of XELOX chemotherapy, postoperatively.

**Figure 3:**
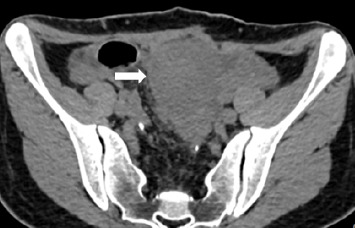
CT-scan showing a large (5.57 × 5.09 cm) intra-abdominal desmoid tumor.

**Figure 4:**
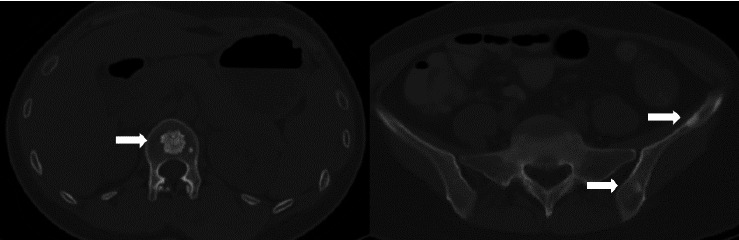
Current CT image of an osteoma in the left ilium and thoracic vertebral bone.

The current preoperative CT examination revealed a soft tissue mass in the pelvis, approximately 5.57∗5.09 cm in size, which was newly developed (Figure 3). Multiple osteomas were found in the thoracic vertebrae T12-L3 and left iliac bone (Figure 4). Anal gastrointestinal tract radiography showed no abnormalities. Whether the newly developed pelvic mass is a tumor recurrence and whether ileostomy closure can be performed remain undetermined.

**Figure 5:**
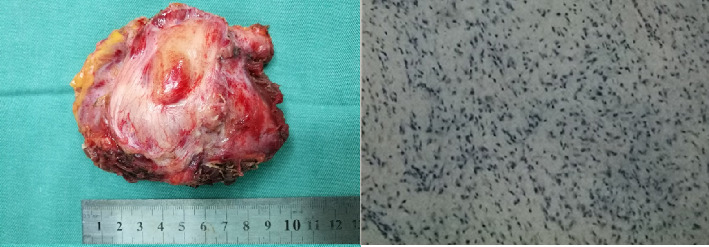
Pathological examination showed that the abdominal wall tumor is an invasive fibroma (hematoxylin and eosin stain; original magnification, 40x).

**Figure 6:**
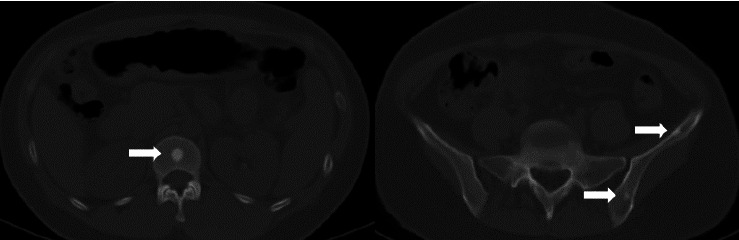
Four-year follow-up CT imaging of an osteoma involving the left left ilium and thoracic vertebral bone.

Four years ago, the patient underwent CT examination for a left abdominal wall mass, which showed a soft tissue density mass in the left rectus sheath, with irregular boundaries and expansile growth, measuring 7.32∗3.5 cm, with uniform internal density. The mass was completely resected surgically with tension-free mesh repair, and postoperative pathology showed aggressive fibromatosis (Figure 5). In addition, CT showed a smaller osteoma than now (Figure 6).

**Figure 7:**
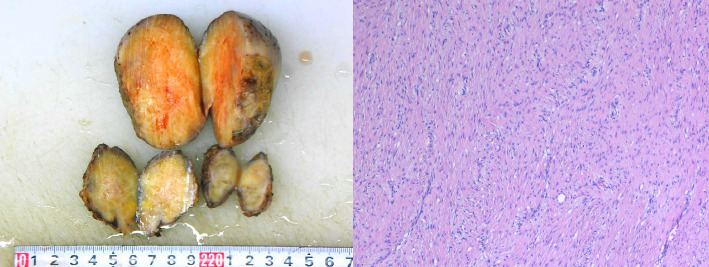
Pathological examination suggests that the mass in the mesentery of the intestines is consistent with invasive fibroma disease (hematoxylin and eosin stain; original magnification, 40x).

Based on the literature review, it is suspected that the patient has a high possibility of GS. The patient underwent resection of the pelvic mass and ileostomy closure, and the postoperative specimen was sent for pathology examination, which indicated mesenteric aggressive fibromatosis (Figure 7).

## 3. Discussion

FAP is a complex genomic syndrome associated with the presence of a large number (exceeding 100) of adenomatous polyps with high malignant potential in the colon. If left untreated, FAP patients almost always develop colorectal cancer (CRC) with a risk close to 100%. FAP patients typically develop CRC approximately by age 40 [4]. Preventive or therapeutic surgeries such as total proctocolectomy with IPAA or total colectomy with ileorectal anastomosis (IRA) are commonly performed for FAP. Therefore, the recent increase in prophylactic colectomies has reduced the risk of FAP patients dying from CRC [5]. In addition, extraintestinal manifestations of FAP have become more clinically significant. As a special manifestation of FAP, GS is characterized by extraintestinal features such as bone tumors, skin tumors, and soft tissue tumors [6]. Osteomas are usually asymptomatic and usually located in the mandible, but may also appear in the cranial bones and long bones, and grow larger over time. Skin tumors include epidermal cysts, lipomas, leiomyomas, and fibromas.

However, through careful examination, it is found that most patients with FAP have at least mild GS manifestations. One such extraintestinal manifestation that has now become a leading cause of death in FAP patients undergoing prophylactic colectomies is DTs [7].

GS is a genetic disorder related to DTs located in the abdominal wall and intra-abdominally. At least 10%–15% of patients with FAP develop DTs that is the second leading cause of death in patients with FAP [8]. The important cause of death in FAP patients is intestinal mesenteric fibromatosis [9], which usually manifests as a slowly growing mass and may lead to symptoms such as intestinal obstruction, enterocutaneous fistula, and hydronephrosis [10]. In addition, having an APC family history, APC gene mutation, a history of abdominal surgery, and being female are risk factors for this disease. Initial management does involve confirming the diagnosis of GS, identifying other affected family members, and reducing the risk of future mortality. To make an accurate diagnosis, doctors typically need to consider multiple factors, including family history, clinical manifestations, intestinal examinations, and genetic testing. As highlighted in this clinical case, reviewing previous medical history of abdominal wall tumors and intestinal tumors, as well as performing further special examinations, can help to diagnose and investigate appropriate treatment methods. These steps are crucial for determining the patient's condition and treatment plan, and can help improve treatment efficacy and patient survival rate.

Complete surgical resection is the primary method for abdominal DT, as DTs within the abdomen often occur at the root of the small intestine mesentery, and extensive bowel resection is often required during surgery. Therefore, nonsurgical treatment, including nonsteroidal anti-inflammatory drugs or antioestrogens, chemotherapy, or radiotherapy, is now more commonly used to treat intra-abdominal DT [11]. However, the rarity and heterogeneity of the disease pose a challenge to the treatment.

## 4. Conclusion

Fibroid development is 800 times more common in people with FAP than in the general population. Early diagnosis of GS is greatly aided by clinicians. Intestinal polyps appear to invariably be preceded by fibroids. Clinical professionals should undertake additional examinations in cases of abnormal bone and body surface disorders, such as osteoma mixed with fibroma, as these conditions may indicate GS.

## Data Availability

All relevant data are within the manuscript and its additional files.
